# Client satisfaction from the perspective of responsiveness: strategy for analysis of universal systems?^1^

**DOI:** 10.1590/1518-8345.1089.2674

**Published:** 2016-03-04

**Authors:** Silvana Martins Mishima, Ana Carolina Campos, Silvia Matumoto, Cinira Magali Fortuna

**Affiliations:** 2 PhD, Full Professor, Escola de Enfermagem de Ribeirão Preto, Universidade de São Paulo, PAHO/WHO Collaborating Centre for Nursing Research Development, Ribeirão Preto, SP, Brazil; 3 MSc, RN, Hospital das Clínicas de Ribeirão Preto, Faculdade de Medicina de Ribeirão Preto, Universidade de São Paulo, Ribeirão Preto, SP, Brazil; 4 PhD, Professor, Escola de Enfermagem de Ribeirão Preto, Universidade de São Paulo, PAHO/WHO Collaborating Centre for Nursing Research Development, Ribeirão Preto, SP, Brazil

**Keywords:** Health Care Quality, Access, and Evaluation, User satisfaction, Primary Health Care, Universal Access to Health Care Services

## Abstract

**Objective:**

to analyze patient satisfaction in a Family Health Unit (FHU) of a municipality in the interior of São Paulo, Brazil, from the perspective of responsiveness.

**Method:**

this was a qualitative study with 41 patients of families who used the FHU at least once in the last six months. A semi-structured interview was used for data collection, performed from November of 2010 to January of 2011, focusing on the dimensions of responsiveness: dignity, autonomy, facilities and physical environment, immediate attention, choice, confidentiality, and communication. A thematic analysis was conducted.

**Results:**

four themes emerged from the analysis: the health unit environment; access and components of accessibility - favoring the responsiveness?; possibilities of developing a patient - health service staff relationship; and the FHU team - processing care and welcoming.

**Conclusion:**

responsiveness allows for the tracking and monitoring of non-medical aspects of care of the patients; it contributes to achieving universal coverage, emphasizing the quality of care.

## Introduction

The concept of responsiveness refers to the way the health system can recognize and respond to the expectations of individuals in relation to non-medical aspects of care, taking into account how government actions meet the expectations and demands of the population, respecting the people's rights by aggregating the principle definition of universal validity to the assessment of health systems (1-4). Thus, responsiveness would review the interaction between the way the health system operates and patient satisfaction^(^ 3 ^)^.

The concept of responsiveness comprises two main aspects: respect for people, which includes questions such as: dignity, autonomy, communication and confidentiality; and customer orientation that includes elements that influence satisfaction, but are not directly connected to health care: respect for rapid attendance, access to social support networks, quality of facilities; and choice of one's health care provider(1,3).

Although criticism exists about the analysis of responsiveness, since it is a patient belief that presents bias, such as gratitude for the care received^(^ 4 ^)^, its elements / dimensions can amplify the possibilities of evaluating patient satisfaction, contributing indicators for expansion of universality of health care and universal coverage. The concept can also enable the nursing team to extend their actions in addressing the needs and expectations of the patients.

Responsiveness is one in ten actions resulting from lessons learned from various Latin American countries that showed that universal coverage is achievable. Such specific action refers to effective mechanisms for monitoring and evaluating quality of care, in the technical and interpersonal dimensions, considering that the expansion of coverage requires investments in the quality of care^(^ 5 ^)^.

Thus, the notion of responsiveness, including the main aspects already mentioned, respect for people and customer orientation, can guide the initiatives that seek to evaluate, monitor and improve quality as a component of universal health coverage^(^ 5 ^)^.

In 1994, the Unified Health System (SUS), in Brazil, had the Family Health Strategy (FHS) implemented and supported by the principles of Primary Health Care (PHC) as a way to address the problem of unequal access and to expand coverage; seeking to accomplish the process of democratization and the development of citizenship, involving the population in decision-making^(^ 6 ^)^.

Studies on health service evaluation (7-8) have indicated the importance of analyzing the impact of PHC actions on the health conditions of the population and patient satisfaction, highlighting the importance of giving them a voice and opportunity to participate in diagnostic processes and action planning. Thus, it is already possible to identify an increase in scientific production on patient satisfaction relating to PHC services, especially those related to Family Health^(^ 8 ^)^. However, studies related to responsiveness remain rare in Brazil.

The difference, from the perspective of responsiveness and evaluation of patient satisfaction, is in its approach when asking: how pleased was the patient with the care received with regard to responsiveness and his expectations, and what happens when he interacts with health services^(^ 4 ^)^.

The research used the dimensions of responsiveness for the analysis of patient satisfaction with the care provided by the Family Health Unit (FHU): dignity, autonomy, confidentiality, immediate attention, choice, facilities. physical environment, and communication

The guiding question of this study was: What is the level of patient satisfaction with the FHU healthcare facilities, considering the constitutive elements of responsiveness?

Thus, the study aimed to analyze patient satisfaction of a FHU from the perspective of responsiveness.

## Methodological framework

This was a descriptive, qualitative study, conducted in a county in São Paulo, Brazil, which has an articulated public network of PHC service provision and different specialized levels of care. In 2011, the city had 21 FHU teams distributed in 13 health care units; and, like other municipalities with more than 100,000 inhabitants, experienced difficulties consolidating the FHS^(^ 9 ^)^, but it was considered important to analyze the results achieved, despite coverage of less than 14%. After presentation of the study, a team was selected by lottery among those who expressed interest in participating in the research.

For data collection, a semi-structured interview allowed for the capture of the singularity and specificities present in the relationship that patients have with the health services, considering the dimensions of responsiveness, which have subjective aspects ^(^ 10 ^)^. The instrument used contained questions relating to the characteristics of the interviewees, and others related to the dimensions of responsiveness, such as: How does the FHU seem to you? Do you feel respected? Can you get care whenever you need it?

The selection of the study subjects was by identification of families registered by the team in the Primary Care Information System (Sistema de Informação de Atenção Básica -SIAB). From a total of 773 families, an initial sample of 77 families (10%) was selected; using simple random sampling without replacement, each sample unit had the same possibility of selection, considering the diversity of the five FHU micro-areas.

Next, from the records of the 77 families, those were identified that had at least one member who had used any of the services offered by the FHU, such as consultations, guidance, home visits, groups, among others, in the period of up to six months prior to September 2, 2010. One member of each selected family, over 18 years of age, present at home on the day and time scheduled for the interview, represented the study subjects. The interviews lasted an average of 45 minutes, and were conducted by experienced interviewers, from November of 2010 to January of 2011. There were audio-recorded and later transcribed into digital media.

Data saturation was verified after 43 interviews, two of which were lost due to recording issues, when the iteration of responses related to the complete dimensions of responsiveness was deduced^(^ 11 ^)^.

Thematic analysis was used for data analysis; its set of procedures allowed the analysis of convergence, divergence and unusual constant answers for open-ended questions (10). The analysis of the empirical material, guided by the responsiveness dimensions, allowed for the identification of four themes: the health unit environment; access and components of accessibility - favoring the responsiveness?; possibilities of developing a patient - health service staff relationship; and the FHU team - processing care and welcoming. 

The study was approved by the Research Ethics Committee, protocol No 400 CEP-CSE-FMRP / USP.

## Resultados

Respondents had diverse opinions about the health unit environment. There are those who considered it appropriate: 

[...] I think it has space enough [indoors], it is shared, well organized there, I think it is good. (Interv.06). 

However, some people asserted that the FHU office is cramped, poorly lit and ventilated, had small corridors with the movement of people hampered, difficult access to stairs, and a small number of offices for medical consultation, namely, they perceive the physical space in the unit is not prepared to promote health care 

[...] the day I went to consult a doctor, I had to wait for the consultation, because they had no office. (Interv.18). 

Patients tend to consider the health unit as a simple place, with old furniture that is inadequate and below their expectations 

[...] but, I think it is an ugly house, moldy, with musty offices, and the reception chairs are not very nice chairs ... (Interv.01); 

and, without proper identification of the FHU on the exterior facade. Additionally, they expressed the wish for a more pleasant and cozy place. 

Some respondents had difficulty reporting on the equipment available: I do not see equipment there [...] (Interv.04). The FHU is seen as a health service with little equipment because is a FHS unit, targeted to monitor and provide promotion and prevention. Complaints from patients about lack of equipment at the FHU were noted: 

[...] I think that they should have more equipment here, that could 

[...] for example, X-Ray. You wait seven to eight hours to get one X-ray by appointment [...] (Interv.04).

And, they believe that it should be better equipped because they spend a lot of time to obtain care at the FHU and then in the District Reference Unit for procedures and tests not available at the FHU.

In the second theme, patients felt there was easy access to the FHU in relation to their residences, with an average of seven minutes of walking 

[...] Oh, where is it, is practically right in the house. Ah, from here to the unit? About three minutes walking [...] (Interv.36). 

However, living close to a health unit does not directly influence access to this service, because the waiting time for scheduling can be long. In this sense, different aspects of organizational accessibility were mentioned, including restriction of places for other levels of care; operating hours of the FHU; waiting time for scheduling appointments; lack of health promotion activities: 

now I miss the PIC [Community Integration Program], because I used to participated in the PIC, it is in the plaza, right, and [...], then, I started to fall, I did not go further [...] (Interv.25).

In relation to the wait time for care at the FHU, is distinguished that it depends on the perspective and needs of each patient, ranging from 15 to 60 minutes, time that is considered appropriate; nearly 90% of the interviewees indicated they received care when they sought it.

The patients indicated dissatisfaction with the accessibility of resources and technology not available at the FHU, such as medications, routine immunizations, urgent and emergency care specific tests and procedures, and specialists

 [...] I think that the FHU should have dental supplies, thus, attending more people [...]. (Interv.16)

In the construction of the relationship between patient - health service staff, confidentiality, which is aspects involving privacy regarding the care provided by the health service and the services performed by the team, the respondents stated that professionals perform assessments, examinations and instructions appropriately. The link with Community Health Agents (CHAs), who visit them in their homes, proved to be important, both because this person is close and is known in the community, as well as due to the confidence transmitted by the professional.

Another aspect of developing confidence in the health service is the confidentiality of information. There were some reports that confidentiality was assured by the team that listens, cares for, guides and treats the person's health, without exposing them to situations of risk and discomfort. Regarding privacy, ethical care was provided with respect, and patients noted safety:

 [...] I think so, there are things I tell them and they would not comment to anyone [...] (Interv.17).

With regard to the theme, The family health team - providing care and welcoming, patients recognize that their desires, problems and needs are heard by the FHU team, emphasizing that the professionals are attentive, careful and sensitive to what is required of them. The average consultation time was positively assessed, ranging from 30 to 60 minutes, because the team did not rush the patient, leaving them free to ask questions and clarify doubts.

Regarding the dimension of choice, more than 90% of respondents denied having a desire to choose another health care facility or other care professional at FHU or for health monitoring of their families, due to the quality of care, close relationship and how they were welcomed and respected 

[...] it is a very close relationship. Well, this thing of family health, because I enter there and the people call me by my name, is a very cool relationship [...] (Interv.21). 

The desire to look for a second opinion was also denied, due to confidence in the care provided by the same team.

Respondents highlighted having the freedom to refuse treatment and guidance, and reported they never had to do this, as the professionals clarified their doubts; they reported they could participate and offer opinions in the decisions about their health and treatment and that of their family. They also stated that the team adequately performed assessments and they felt supported, cared for and confident in the prescriptions and guidance offered, favoring the patient-health service link: 

Ah! I think the professionals do the best they can [...] think they like what they do, are highly motivated [...] (Interv.31)

The study participants explained that the professionals who treated them always asked what bothered them, and they never ceased to clarify things, stating that the health care team was available for guidance and information. They felt that appropriate care went beyond the team's commitment with the care itself, but resulted from the bond built on the patient-professionals - health facility triad.

## Discussion

The PHC in Brazil prioritized the FHS implementing necessary changes with a view to consolidating the principles of the Unified Health System ^(^ 7 ^,^ 9 ^)^, making effective efforts to improve coverage, thereby meeting the health needs of population^(^ 6 ^)^.

The FHU should be structured to accommodate teams that can count on a working process, with structural resources and devices compatible with the necessary health actions ^(^ 12 ^)^ to face the challenges imposed on the health systems^(^ 6 ^)^. In the case discussed here, this refers to the organization of health services in order to include the expansion of coverage, with quality of care^(^ 5 ^)^.

This movement also includes the perspective of systems to offer comprehensive and integrated care, at all levels. Such care needs to be monitored and evaluated, institutionally and by the patients themselves, who now have a fundamental role in assessing the ability of the services to satisfy their needs and expectations^(^ 13 ^)^.

Responsiveness and its main aspects enabled us to present the evaluation of the FHU by participants of the study.

Regarding the *respect for people*, the patients value the "courtesy" which is considered a soft technology, namely a technology of relationship, in which inter-subjectivity operates in the act of producing health actions, interaction processes, bonding, and listening, which have also been mentioned as fundamental in other studies^(^ 12 ^,^ 14 ^)^.

The dignity in care dimension was analyzed and listed as a respectful form of caring, with the welcoming by the staff without offense and that does not adversely affect the human rights of citizens and health clients. In terms of human rights, dignity implies a set of rights and a guarantee, in which all individuals shall be treated with respect and remain free to achieve their own dreams and hopes. The most important way to ensure human dignity is the fight against unjust discrimination based on race, sex, religion, ethnicity, political opinion, property, disability or other status. The right to health is closely linked to and dependent upon the realization of other human rights, such as the right to food, housing, work, education, human dignity, life, non-discrimination, equality, the prohibition against torture, privacy, access to information, and freedom of choice and expression^(^ 6 ^)^. The principles of human rights are internationally recognized and accepted, and are composed of civil, cultural, economic, political and social rights. In this manner, the quest for universal coverage led some Latin American countries to adopt health as a social and citizenship right^(^ 5 ^)^.

Each one of the responsiveness aspects are supported by one or more human rights principles, and these aspects are interconnected with each other. The care provided with respect, caring and dignity involves patient autonomy to participate and decide on one's own health and that of one's family, as well as the freedom to accept or refuse treatment and health guidance. Regarding privacy and confidentiality,^(^ 12 ^)^ patients felt protected in the service evaluated, unlike those in the services of Rio de Janeiro, whose clients evaluated the former as the most flawed aspect, although the confidentiality of information was assured^(^ 4 ^)^.

Communication was perceived in the construction of responsiveness^(^ 1 ^)^ as the point common to all other aspects of this theme, and it was fundamental to the continuity of care. The quality of care depends on the combination of technical and interpersonal measures, namely, proper and effective communication with language that favors the understanding of the patients, as reported by the interviewees, reiterating satisfaction with the explanations received. In this sense, the service moves in the direction of strengthening the health system functions, to enlarge the choice of the patient, increasing effectiveness and efficiency, as well as improving the quality of care, aiming at universal coverage ^(5)^. In addition, for patients of health services, the expectations are built by the means of communication based on the current and past experiences involving values, beliefs and attitudes regarding the rights and duties of citizenship ^(^ 1 ^,^ 8 ^)^. 

The other set of responsiveness aspects refers to customer orientation. One of the elements includes facilities and the physical environment. Thus, it appears that this dimension was poorly judged by FHU patients, shown to be inadequate and not conforming to the wishes and expectations of the population; however, this aspect is minimized when the issue is the quality of care provided in the unit.

Studies^(^ 8 ^,^ 12 ^,^ 15 ^-^ 17 ^)^ show that a majority of the FHS units had inadequate and/or improvised physical structure, or had difficulty maintaining a flow for referral services, or even insufficiency support services (equipment, medications, etc.). These facts may suggest a lack of appreciation by the local authorities about these services, given the lack of investment to enable the structuring of services to change the model of care. Additionally, these services are evaluated as low cost, and focus on care for the poorest populations or in places where there is no installed health equipment. Accessibility in health is linked to the quality of care provided to the population, and "allows for the identification of factors that facilitate or hinder the seeking and obtaining of this assistance"^(^ 16 ^)^.

The importance of the adequacy of appropriate space for care, with good lighting, ventilation and absence of noise, as well as other aspects that help to provide conditions necessary to ensure the comfort and confidentiality of care are fundamental, ensuring the privacy of patients. In services, the needs of hospitalized patients or PHC units are not considered, or where there is scarcity of material resources, environments that offer no room to accommodate the social network of patients, and other care that assists in the preservation of their identities, worsening and increasing of patient vulnerability can be verified, causing discomfort, concern, insecurity and embarrassment with the care provided^(^ 14 ^)^. Thus, the health care facility environment can not harm the integrity of patients, but rather, must provide a more welcoming space for the ills and sufferings that the patient expresses^(^ 14 ^-^ 15 ^,^ 17 ^)^.

The immediate care dimension was explored from two perspectives. The waiting time for care dimension was satisfactory in terms of user expectation. In research on services in Rio de Janeiro, 40% of respondents were unhappy with waiting more than one hour ^(^ 4 ^)^. The other perspective of immediate care (or rapid attendance) with regard to geographic accessibility, defined as the "dimension that reflects the average distance between population and resources and should be measured in terms of time consumed for health care, by the usual means of transportation." . 

It is not possible to set an ideal measure of geographical accessibility, as adequate time for analysis depends on the type of need. [...] There are different levels of accessibility for different needs, which, in turn, should be covered by different resource characteristics" ^(^ 16 ^)^.

As stated, the proximity of the unit (geographical accessibility) does not always mean accessibility to the unit. Other dimensions such as organizational accessibility, are part of the service's response to the users' needs. In this sense, the operation of the FHU during working hours was considered inappropriate, as it is inconvenient for service workers. Other aspects, such as those related to obstacles to continuity of care at other levels, linked to the hierarchical level of network care ^(^ 16 ^)^, namely, those related to referral and counter-referral, also hinder the achievement of universal access.

Also, the patient-worker relationship is "prepared" much before they meet, including the service aim, work execution, environment structuring and others. In terms of the environment, it should be prepared as relational space, where people try to solve problems in an appropriate and decisive manner ^(^ 14 ^)^. The preparation of the physical space goes beyond architectural planning, but also ensures that the patient feels safe and respected to express his problems and needs ^(^ 14 ^)^. For the patients, aspects related to infrastructure of the FHU compromise the way the population perceives the health unit, and how they can expose their health needs with less embarrassment, and with more safety, reliability, and comfort^(^ 12 ^,^ 14 ^,^ 17 ^)^. The relationship, a well maintained environment, and health seem to be directly linked to seeking out health services and the expectations of the population for dignified, caring and effective care.

This discussion on responsiveness provides elements present in the relationship of the patient with the health services, mediated by a team that needs to have instruments to understand the patient's health needs and to program actions that enable the expression of: patient freedom for adhering to the treatment plan that the physician and/or team propose, with full patient participation in decisions about his own health.

## Final considerations

The construction of patient satisfaction assessment tools to analyze social and cultural differences, and different ways of using the services, is a new and challenging practice, because of the need to capture the views of patients about the quality of health services, and also, this depends on resources and commitment so that monitoring can be consistently performed and which enable effective improvements in services, as well as the expansion of universal access.

Responsiveness organizes elements already explored as components of user satisfaction, highlighting the non-medical aspects of care that reveal the other side of quality of care. This proposes to track and monitor government actions to meet the population's needs and expectations, converging with actions recognized as positive for achieving universal health coverage.

The results allow us to state advances in the assessment of the Family Health Strategy as a health service that is close and accessible to the population, which welcomes, cares for and monitors the health of patients and their families, and creates bonds with the population, through attentive and caring assistance with their needs and desires. This ensures participation and freedom for decisions and choice of treatments, ensuring confidentiality and privacy.

The study had the limitation of being conducted with only one FHU, in the context of low total coverage by the FHS, thus, having some bias resulting from this broader context of the municipality. Still, it contributes an assessment tool for patient satisfaction, which helps with the expansion of universal health coverage.
